# Bis[*N*-(2-pyridylcarbon­yl)pyridine-2-carboximidato]iron(III) perchlorate methanol solvate

**DOI:** 10.1107/S1600536809040549

**Published:** 2009-10-10

**Authors:** Dayu Wu

**Affiliations:** a Anhui Key Laboratory of Functional Coordination Compounds, School of Chemistry and Chemical Engineering, Anqing Teachers College, Anqing, 246011 Anhui, People’s Republic of China

## Abstract

In the title complex, [Fe(C_12_H_8_N_3_O_2_)_2_]ClO_4_·CH_3_OH, the iron(III) ion is surrounded by two tridentate *N*-(2-pyridyl­carbon­yl)pyridine-2-carboximidate (bpca) ligands and exhib­its a distorted octa­hedral coordination by six bpca N atoms. A classical O—H⋯O hydrogen bond exists between the methanol solvent mol­ecule and the perchlorate anion. Magnetic susceptibility measurements indicated the complex to be in the low-spin state in the temperature range 5–400 K.

## Related literature

For the structure and magnetic properties of methanol-free [Fe(bpca)_2_]ClO_4_ and related compounds, see: Wocadlo *et al.* (1993 ▶).
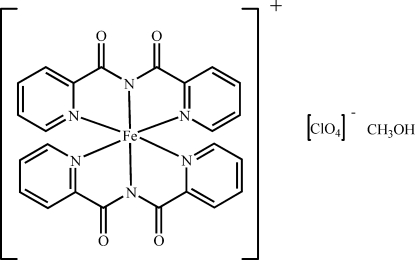


## Experimental

### 

#### Crystal data

[Fe(C_12_H_8_N_3_O_2_)_2_]ClO_4_·CH_4_O
*M* *_r_* = 639.77
Triclinic, 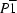

*a* = 8.799 (3) Å
*b* = 11.603 (4) Å
*c* = 14.356 (6) Å
α = 109.507 (4)°
β = 103.394 (3)°
γ = 100.091 (3)°
*V* = 1292.0 (8) Å^3^
*Z* = 2
Mo *K*α radiation
μ = 0.76 mm^−1^
*T* = 143 K
0.32 × 0.26 × 0.23 mm

#### Data collection

Bruker SMART APEX CCD area-detector diffractometer
Absorption correction: multi-scan(*SHELXTL*; Sheldrick, 2008 ▶) *T*_min_ = 0.790, *T*_max_ = 0.840
8499 measured reflections
4421 independent reflections
4177 reflections with *I* > 2σ(*I*)
*R*_int_ = 0.040

#### Refinement

*R*[*F* ^2^ > 2σ(*F* ^2^)] = 0.040
*wR*(*F* ^2^) = 0.107
*S* = 1.03
4421 reflections
379 parameters
H-atom parameters constrained
Δρ_max_ = 1.48 e Å^−3^
Δρ_min_ = −0.48 e Å^−3^

### 

Data collection: *SMART* (Bruker, 1997 ▶); cell refinement: *SAINT* (Bruker, 1997 ▶); data reduction: *SAINT*; program(s) used to solve structure: *SHELXS97* (Sheldrick, 2008 ▶); program(s) used to refine structure: *SHELXL97* (Sheldrick, 2008 ▶); molecular graphics: *SHELXTL* (Sheldrick, 2008 ▶); software used to prepare material for publication: *SHELXL97* and *PLATON* (Spek, 2009 ▶).

## Supplementary Material

Crystal structure: contains datablocks I, global. DOI: 10.1107/S1600536809040549/si2207sup1.cif

Structure factors: contains datablocks I. DOI: 10.1107/S1600536809040549/si2207Isup2.hkl

Additional supplementary materials:  crystallographic information; 3D view; checkCIF report

## Figures and Tables

**Table 1:** Selected bond lengths (Å)

Fe1—N2	1.900 (2)
Fe1—N5	1.922 (2)
Fe1—N6	1.974 (2)
Fe1—N4	1.976 (2)
Fe1—N1	1.977 (2)
Fe1—N3	1.977 (2)

**Table 2:** Hydrogen-bond geometry (Å, °)

*D*—H⋯*A*	*D*—H	H⋯*A*	*D*⋯*A*	*D*—H⋯*A*
O1*W*—H1*W*⋯O13^i^	1.03	1.92	2.916 (3)	160

Symmetry code: (i) 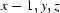.

**Fig. 1.**
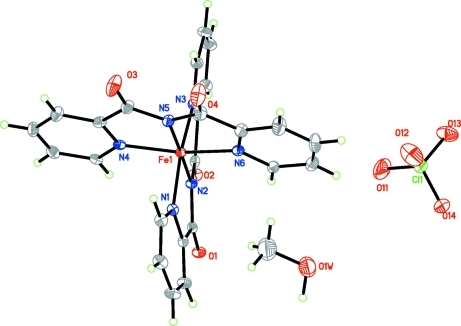
The molecular structure of the title compound, the thermal ellipsoids were drawn at 50% probability level.

## supplementary crystallographic information

### Comment

Our recent work indicated the N-donor tridentate ligand is suitable for the synthesis of spin-crossover materials. The *N*-2-pyridinylcarbonyl-2-pyridinecarboximidate (bpca) ligand has a typical rigid tridentate donor and was well studied to construct transition metal complexes inculding Fe(II), Fe(III), Co(II), Ni(II) and Cu(II) (Wocadlo *et al.* 1993 and references cited therein).. One of the examples is reported by Wocadlo and coworkers, which interestingly showed the spin state can be tuned by the different counterion and solvent. It was claimed that Fe(III) complex [Fe(bpca)Cl_2_(H_2_O)] (CH_3_)_2_CO and [Fe(bpca)_2_](NO_3_) 1.67 H_2_O adopt high spin state and the low-spin one in all the range of measured temperatures, respectively, while the [Fe(bpca)_2_](C1O_4_) evidence the spin-crossover behaviour. Here, we reported the crystal stucture of complex [Fe(bpca)_6_](C1O_4_) CH_3_OH.(Fig. 1). The coordination environments of Fe(III) ions are completed by two bpca ligands with average Fe—N bond length of being 1.954 Å (Table 1). A classical hyrogen bond O—H···O exists between methanol and chlorate anion with D···A distance being 2.916 (3)Å (Table 2). The temperature-dependent magnetic susceptibility was measured down to 5 K. The data in the form of molar magnetic moment multiply temperature is nearly constant and equal to about 0.45 emu K mol^-1^, consistent with low spin state of Fe(III) (s = 1/2).

### Experimental

A methanolic solution (25 ml) containing the bpca ligand (0.2 mmol, 0.046 g) was added dropwise to Fe(ClO_4_)_2_.6 H_2_O (0.1 mmol, 0.036 g). After stirring for 15 minutes, the dark solution was filtered. Red block-shaped crystals suitable for single-crystal X-ray diffraction were obtained by evaporating the resulting filtration in air for several days (yield: 56.2%). Anal calc (%). for C_25_ H_20_ Cl Fe N_6_ O_9_: H 3.15 C 46.95 N 13.15. Found: H 3.12, C 46.87, N 13.54.

### Refinement

C-bound H atoms were placed geometrically and allowed to ride during refinement with C—H = 0.93–0.96 Å with *U*_iso_(H) = 1.2 *U*_eq_(C). The hydroxy H atom of the methanol solvent molecule was located in a difference Fourier map and refined as riding with the parent atom with *U*_iso_(H) = 1.5*U*_eq_(O).

### Crystal data

**Table:**

[Fe(C_12_H_8_N_3_O_2_)_2_]ClO_4_·CH_4_O	*Z* = 2
*M**_r_* = 639.77	*F*(000) = 654
Triclinic, *P*1	*D*_x_ = 1.645 Mg m^−^^3^
Hall symbol: -P 1	Mo *K*α radiation, λ = 0.71070 Å
*a* = 8.799 (3) Å	Cell parameters from 5465 reflections
*b* = 11.603 (4) Å	θ = 3.0–27.8°
*c* = 14.356 (6) Å	µ = 0.76 mm^−^^1^
α = 109.507 (4)°	*T* = 143 K
β = 103.394 (3)°	Block, red
γ = 100.091 (3)°	0.32 × 0.26 × 0.23 mm
*V* = 1292.0 (8) Å^3^	

### Data collection

**Table:**

Bruker SMART APEX CCD area-detector diffractometer	4421 independent reflections
Radiation source: fine-focus sealed tube	4177 reflections with *I* > 2σ(*I*)
graphite	*R*_int_ = 0.040
φ and ω scans	θ_max_ = 25.0°, θ_min_ = 3.2°
Absorption correction: multi-scan (*SHELXTL*; Sheldrick, 2008)	*h* = −8→10
*T*_min_ = 0.790, *T*_max_ = 0.840	*k* = −13→13
8499 measured reflections	*l* = −17→16

### Refinement

**Table:**

Refinement on *F*^2^	Primary atom site location: structure-invariant direct methods
Least-squares matrix: full	Secondary atom site location: difference Fourier map
*R*[*F*^2^ > 2σ(*F*^2^)] = 0.040	Hydrogen site location: inferred from neighbouring sites
*wR*(*F*^2^) = 0.107	H-atom parameters constrained
*S* = 1.03	*w* = 1/[σ^2^(*F*_o_^2^) + (0.0537*P*)^2^ + 1.8601*P*] where *P* = (*F*_o_^2^ + 2*F*_c_^2^)/3
4421 reflections	(Δ/σ)_max_ < 0.001
379 parameters	Δρ_max_ = 1.48 e Å^−^^3^
0 restraints	Δρ_min_ = −0.48 e Å^−^^3^

### Special details

**Table:**

Experimental. The magnetic measurements were performed on Quantum Design SQUID, MPMS-5S magnetometer.
Geometry. All e.s.d.'s (except the e.s.d. in the dihedral angle between two l.s. planes) are estimated using the full covariance matrix. The cell e.s.d.'s are taken into account individually in the estimation of e.s.d.'s in distances, angles and torsion angles; correlations between e.s.d.'s in cell parameters are only used when they are defined by crystal symmetry. An approximate (isotropic) treatment of cell e.s.d.'s is used for estimating e.s.d.'s involving l.s. planes.
Refinement. Refinement of *F*^2^ against ALL reflections. The weighted *R*-factor *wR* and goodness of fit *S* are based on *F*^2^, conventional *R*-factors *R* are based on *F*, with *F* set to zero for negative *F*^2^. The threshold expression of *F*^2^ > σ(*F*^2^) is used only for calculating *R*-factors(gt) *etc*. and is not relevant to the choice of reflections for refinement. *R*-factors based on *F*^2^ are statistically about twice as large as those based on *F*, and *R*- factors based on ALL data will be even larger.

### Fractional atomic coordinates and isotropic or equivalent isotropic displacement parameters (Å^2^)

**Table:**

	*x*	*y*	*z*	*U*_iso_*/*U*_eq_	
Fe1	0.58016 (4)	0.81056 (3)	0.23045 (2)	0.01007 (12)	
O1	0.2790 (2)	0.51858 (16)	−0.02782 (13)	0.0148 (4)	
N2	0.5013 (2)	0.68770 (19)	0.09138 (15)	0.0112 (4)	
O2	0.5569 (2)	0.63892 (17)	−0.06690 (13)	0.0168 (4)	
N1	0.3602 (2)	0.73605 (19)	0.23053 (15)	0.0121 (4)	
N6	0.6689 (2)	0.70815 (19)	0.30342 (16)	0.0129 (4)	
N3	0.7802 (2)	0.84703 (19)	0.19255 (16)	0.0127 (4)	
N5	0.6579 (2)	0.93790 (19)	0.36992 (15)	0.0136 (4)	
N4	0.5149 (2)	0.95113 (19)	0.19857 (15)	0.0124 (4)	
C5	0.2725 (3)	0.6311 (2)	0.14314 (18)	0.0118 (5)	
O4	0.7774 (3)	0.97129 (19)	0.54311 (14)	0.0338 (5)	
O3	0.6870 (3)	1.15149 (18)	0.46500 (15)	0.0326 (5)	
C7	0.5952 (3)	0.6937 (2)	0.02702 (18)	0.0121 (5)	
C6	0.3479 (3)	0.6029 (2)	0.05672 (18)	0.0119 (5)	
C9	0.8828 (3)	0.7988 (2)	0.0474 (2)	0.0175 (5)	
H9A	0.8652	0.7542	−0.0230	0.021*	
C4	0.1249 (3)	0.5577 (2)	0.13335 (19)	0.0165 (5)	
H4A	0.0705	0.4844	0.0739	0.020*	
C15	0.4363 (3)	1.1664 (3)	0.1802 (2)	0.0202 (6)	
H15A	0.4089	1.2379	0.1738	0.024*	
C13	0.4368 (3)	0.9487 (2)	0.10510 (19)	0.0141 (5)	
H13A	0.4096	0.8740	0.0464	0.017*	
C8	0.7602 (3)	0.7837 (2)	0.09035 (19)	0.0133 (5)	
C11	1.0553 (3)	0.9449 (3)	0.2156 (2)	0.0211 (6)	
H11A	1.1562	0.9995	0.2597	0.025*	
C10	1.0327 (3)	0.8812 (3)	0.1110 (2)	0.0226 (6)	
H10A	1.1174	0.8938	0.0838	0.027*	
C24	0.6699 (3)	0.5865 (2)	0.2595 (2)	0.0145 (5)	
H24A	0.6253	0.5434	0.1878	0.017*	
C14	0.3960 (3)	1.0545 (3)	0.0939 (2)	0.0178 (5)	
H14A	0.3417	1.0501	0.0285	0.021*	
C22	0.8026 (4)	0.5867 (3)	0.4249 (2)	0.0273 (6)	
H22A	0.8482	0.5458	0.4653	0.033*	
C17	0.5555 (3)	1.0616 (2)	0.28249 (19)	0.0160 (5)	
C18	0.6414 (3)	1.0583 (2)	0.3844 (2)	0.0182 (5)	
C3	0.0577 (3)	0.5945 (3)	0.2137 (2)	0.0189 (5)	
H3A	−0.0422	0.5463	0.2089	0.023*	
C20	0.7334 (3)	0.7695 (2)	0.40816 (19)	0.0177 (5)	
C21	0.8008 (4)	0.7121 (3)	0.4710 (2)	0.0257 (6)	
H21A	0.8441	0.7564	0.5427	0.031*	
C1	0.2943 (3)	0.7719 (2)	0.30753 (19)	0.0157 (5)	
H1A	0.3519	0.8443	0.3672	0.019*	
C12	0.9269 (3)	0.9267 (2)	0.25399 (19)	0.0156 (5)	
H12A	0.9423	0.9708	0.3242	0.019*	
C23	0.7360 (3)	0.5236 (3)	0.3187 (2)	0.0205 (6)	
H23A	0.7352	0.4393	0.2868	0.025*	
C19	0.7269 (3)	0.9048 (2)	0.4504 (2)	0.0188 (5)	
C16	0.5182 (3)	1.1700 (3)	0.2764 (2)	0.0208 (6)	
H16A	0.5476	1.2442	0.3358	0.025*	
C2	0.1426 (3)	0.7039 (3)	0.3005 (2)	0.0193 (6)	
H2A	0.0985	0.7320	0.3542	0.023*	
Cl1	0.91561 (7)	0.22764 (6)	0.18640 (5)	0.02052 (17)	
O14	0.8085 (2)	0.10315 (19)	0.15308 (18)	0.0319 (5)	
O13	1.0750 (2)	0.2169 (2)	0.18132 (17)	0.0346 (5)	
O12	0.9291 (3)	0.3020 (2)	0.29168 (18)	0.0458 (6)	
O11	0.8522 (3)	0.2870 (2)	0.1189 (2)	0.0512 (7)	
C1W	0.3474 (5)	0.5122 (4)	0.3889 (3)	0.0481 (9)	
H1WA	0.2545	0.5255	0.4108	0.072*	
H1WB	0.3419	0.5313	0.3283	0.072*	
H1WC	0.4448	0.5668	0.4438	0.072*	
O1W	0.3486 (3)	0.3837 (2)	0.36481 (19)	0.0415 (6)	
H1W	0.2370	0.3263	0.3121	0.050*	

### Atomic displacement parameters (Å^2^)

**Table:**

	*U*^11^	*U*^22^	*U*^33^	*U*^12^	*U*^13^	*U*^23^
Fe1	0.01026 (19)	0.0101 (2)	0.0073 (2)	0.00144 (14)	0.00143 (14)	0.00187 (15)
O1	0.0143 (8)	0.0139 (9)	0.0091 (9)	0.0001 (7)	0.0000 (7)	0.0002 (8)
N2	0.0109 (10)	0.0113 (10)	0.0095 (10)	0.0014 (8)	0.0027 (8)	0.0027 (8)
O2	0.0202 (9)	0.0179 (9)	0.0092 (9)	0.0018 (7)	0.0047 (7)	0.0033 (7)
N1	0.0133 (10)	0.0139 (10)	0.0091 (10)	0.0041 (8)	0.0033 (8)	0.0045 (9)
N6	0.0126 (10)	0.0131 (10)	0.0121 (10)	0.0022 (8)	0.0044 (8)	0.0042 (9)
N3	0.0124 (10)	0.0123 (10)	0.0122 (10)	0.0022 (8)	0.0014 (8)	0.0056 (8)
N5	0.0160 (10)	0.0115 (10)	0.0091 (10)	0.0025 (8)	0.0011 (8)	0.0015 (8)
N4	0.0097 (9)	0.0148 (10)	0.0126 (10)	0.0012 (8)	0.0046 (8)	0.0054 (9)
C5	0.0137 (11)	0.0116 (11)	0.0095 (11)	0.0045 (9)	0.0029 (9)	0.0033 (10)
O4	0.0575 (15)	0.0241 (11)	0.0087 (10)	0.0165 (10)	−0.0048 (9)	0.0003 (9)
O3	0.0580 (14)	0.0145 (10)	0.0134 (10)	0.0100 (9)	−0.0006 (9)	−0.0009 (9)
C7	0.0141 (12)	0.0115 (12)	0.0127 (13)	0.0056 (9)	0.0053 (10)	0.0053 (10)
C6	0.0125 (11)	0.0122 (12)	0.0113 (12)	0.0041 (9)	0.0020 (10)	0.0057 (11)
C9	0.0177 (12)	0.0204 (13)	0.0164 (13)	0.0053 (10)	0.0068 (10)	0.0086 (11)
C4	0.0143 (12)	0.0179 (13)	0.0117 (12)	0.0012 (10)	0.0010 (10)	0.0028 (10)
C15	0.0205 (13)	0.0209 (14)	0.0277 (15)	0.0101 (11)	0.0104 (11)	0.0157 (12)
C13	0.0098 (11)	0.0188 (13)	0.0123 (12)	0.0019 (9)	0.0026 (9)	0.0058 (10)
C8	0.0140 (12)	0.0139 (12)	0.0132 (12)	0.0049 (10)	0.0035 (10)	0.0067 (10)
C11	0.0116 (12)	0.0226 (14)	0.0256 (15)	0.0007 (10)	0.0006 (11)	0.0108 (12)
C10	0.0143 (12)	0.0304 (15)	0.0271 (15)	0.0053 (11)	0.0080 (11)	0.0156 (13)
C24	0.0145 (11)	0.0134 (12)	0.0163 (12)	0.0045 (9)	0.0079 (10)	0.0043 (10)
C14	0.0136 (12)	0.0257 (14)	0.0194 (13)	0.0061 (10)	0.0066 (10)	0.0140 (12)
C22	0.0356 (16)	0.0287 (16)	0.0272 (16)	0.0166 (13)	0.0088 (13)	0.0192 (13)
C17	0.0147 (12)	0.0156 (12)	0.0147 (13)	0.0010 (10)	0.0031 (10)	0.0048 (11)
C18	0.0219 (13)	0.0141 (13)	0.0150 (13)	0.0038 (10)	0.0032 (10)	0.0036 (11)
C3	0.0127 (12)	0.0250 (14)	0.0169 (13)	0.0005 (10)	0.0048 (10)	0.0079 (11)
C20	0.0204 (13)	0.0175 (13)	0.0132 (13)	0.0045 (10)	0.0032 (10)	0.0054 (11)
C21	0.0362 (16)	0.0251 (15)	0.0149 (13)	0.0113 (12)	0.0030 (12)	0.0087 (12)
C1	0.0178 (12)	0.0176 (13)	0.0112 (12)	0.0057 (10)	0.0048 (10)	0.0045 (10)
C12	0.0152 (12)	0.0156 (12)	0.0131 (12)	0.0023 (10)	0.0002 (10)	0.0060 (10)
C23	0.0229 (13)	0.0172 (13)	0.0268 (15)	0.0077 (11)	0.0144 (12)	0.0098 (12)
C19	0.0218 (13)	0.0180 (13)	0.0120 (13)	0.0058 (11)	0.0004 (10)	0.0036 (11)
C16	0.0260 (14)	0.0148 (13)	0.0210 (14)	0.0067 (11)	0.0078 (11)	0.0055 (11)
C2	0.0194 (13)	0.0269 (15)	0.0153 (13)	0.0084 (11)	0.0093 (11)	0.0091 (12)
Cl1	0.0209 (3)	0.0169 (3)	0.0216 (3)	0.0046 (3)	0.0049 (3)	0.0063 (3)
O14	0.0243 (10)	0.0215 (11)	0.0485 (13)	0.0023 (8)	0.0087 (10)	0.0160 (10)
O13	0.0217 (10)	0.0423 (13)	0.0359 (12)	0.0036 (9)	0.0139 (9)	0.0097 (11)
O12	0.0401 (13)	0.0555 (16)	0.0267 (12)	0.0222 (12)	0.0075 (10)	−0.0049 (11)
O11	0.0555 (16)	0.0345 (13)	0.0552 (16)	0.0034 (12)	−0.0092 (13)	0.0299 (12)
C1W	0.049 (2)	0.049 (2)	0.055 (2)	0.0203 (18)	0.0172 (18)	0.0263 (19)
O1W	0.0393 (13)	0.0343 (13)	0.0425 (14)	0.0149 (10)	0.0025 (11)	0.0092 (11)

### Geometric parameters (Å, °)

**Table:**

Fe1—N2	1.900 (2)	C13—H13A	0.9300
Fe1—N5	1.922 (2)	C11—C10	1.381 (4)
Fe1—N6	1.974 (2)	C11—C12	1.381 (4)
Fe1—N4	1.976 (2)	C11—H11A	0.9300
Fe1—N1	1.977 (2)	C10—H10A	0.9300
Fe1—N3	1.977 (2)	C24—C23	1.388 (4)
O1—C6	1.206 (3)	C24—H24A	0.9300
N2—C7	1.384 (3)	C14—H14A	0.9300
N2—C6	1.391 (3)	C22—C23	1.377 (4)
O2—C7	1.216 (3)	C22—C21	1.387 (4)
N1—C1	1.344 (3)	C22—H22A	0.9300
N1—C5	1.362 (3)	C17—C16	1.379 (4)
N6—C24	1.343 (3)	C17—C18	1.500 (4)
N6—C20	1.356 (3)	C3—C2	1.380 (4)
N3—C12	1.345 (3)	C3—H3A	0.9300
N3—C8	1.356 (3)	C20—C21	1.379 (4)
N5—C19	1.379 (3)	C20—C19	1.500 (4)
N5—C18	1.381 (3)	C21—H21A	0.9300
N4—C13	1.347 (3)	C1—C2	1.390 (4)
N4—C17	1.353 (3)	C1—H1A	0.9300
C5—C4	1.371 (3)	C12—H12A	0.9300
C5—C6	1.507 (3)	C23—H23A	0.9300
O4—C19	1.219 (3)	C16—H16A	0.9300
O3—C18	1.211 (3)	C2—H2A	0.9300
C7—C8	1.499 (3)	Cl1—O12	1.433 (2)
C9—C8	1.374 (4)	Cl1—O14	1.434 (2)
C9—C10	1.380 (4)	Cl1—O11	1.434 (2)
C9—H9A	0.9300	Cl1—O13	1.446 (2)
C4—C3	1.392 (4)	C1W—O1W	1.416 (4)
C4—H4A	0.9300	C1W—H1WA	0.9600
C15—C14	1.381 (4)	C1W—H1WB	0.9600
C15—C16	1.384 (4)	C1W—H1WC	0.9600
C15—H15A	0.9300	O1W—H1W	1.0342
C13—C14	1.385 (4)		
			
N2—Fe1—N5	178.55 (8)	C12—C11—H11A	120.3
N2—Fe1—N6	100.02 (9)	C9—C10—C11	119.2 (2)
N5—Fe1—N6	81.43 (9)	C9—C10—H10A	120.4
N2—Fe1—N4	96.55 (9)	C11—C10—H10A	120.4
N5—Fe1—N4	82.01 (9)	N6—C24—C23	121.5 (2)
N6—Fe1—N4	163.43 (9)	N6—C24—H24A	119.2
N2—Fe1—N1	82.14 (8)	C23—C24—H24A	119.2
N5—Fe1—N1	97.85 (9)	C15—C14—C13	119.8 (2)
N6—Fe1—N1	89.91 (8)	C15—C14—H14A	120.1
N4—Fe1—N1	92.51 (8)	C13—C14—H14A	120.1
N2—Fe1—N3	82.31 (8)	C23—C22—C21	119.2 (3)
N5—Fe1—N3	97.73 (9)	C23—C22—H22A	120.4
N6—Fe1—N3	91.22 (8)	C21—C22—H22A	120.4
N4—Fe1—N3	90.84 (8)	N4—C17—C16	122.9 (2)
N1—Fe1—N3	164.37 (9)	N4—C17—C18	115.5 (2)
C7—N2—C6	123.1 (2)	C16—C17—C18	121.6 (2)
C7—N2—Fe1	117.82 (16)	O3—C18—N5	127.9 (2)
C6—N2—Fe1	118.93 (16)	O3—C18—C17	122.0 (2)
C1—N1—C5	118.1 (2)	N5—C18—C17	110.1 (2)
C1—N1—Fe1	127.89 (17)	C2—C3—C4	118.6 (2)
C5—N1—Fe1	113.91 (16)	C2—C3—H3A	120.7
C24—N6—C20	118.5 (2)	C4—C3—H3A	120.7
C24—N6—Fe1	126.44 (17)	N6—C20—C21	122.6 (2)
C20—N6—Fe1	115.01 (17)	N6—C20—C19	114.9 (2)
C12—N3—C8	118.2 (2)	C21—C20—C19	122.6 (2)
C12—N3—Fe1	128.32 (17)	C20—C21—C22	118.5 (3)
C8—N3—Fe1	113.43 (16)	C20—C21—H21A	120.7
C19—N5—C18	123.3 (2)	C22—C21—H21A	120.7
C19—N5—Fe1	118.66 (17)	N1—C1—C2	121.8 (2)
C18—N5—Fe1	118.07 (16)	N1—C1—H1A	119.1
C13—N4—C17	117.9 (2)	C2—C1—H1A	119.1
C13—N4—Fe1	127.82 (17)	N3—C12—C11	121.8 (2)
C17—N4—Fe1	114.28 (16)	N3—C12—H12A	119.1
N1—C5—C4	122.6 (2)	C11—C12—H12A	119.1
N1—C5—C6	115.1 (2)	C22—C23—C24	119.7 (3)
C4—C5—C6	122.3 (2)	C22—C23—H23A	120.2
O2—C7—N2	128.2 (2)	C24—C23—H23A	120.2
O2—C7—C8	122.1 (2)	O4—C19—N5	127.6 (2)
N2—C7—C8	109.7 (2)	O4—C19—C20	122.4 (2)
O1—C6—N2	128.2 (2)	N5—C19—C20	110.0 (2)
O1—C6—C5	122.5 (2)	C17—C16—C15	118.8 (2)
N2—C6—C5	109.3 (2)	C17—C16—H16A	120.6
C8—C9—C10	118.7 (2)	C15—C16—H16A	120.6
C8—C9—H9A	120.6	C3—C2—C1	119.7 (2)
C10—C9—H9A	120.6	C3—C2—H2A	120.2
C5—C4—C3	119.1 (2)	C1—C2—H2A	120.2
C5—C4—H4A	120.5	O12—Cl1—O14	109.89 (15)
C3—C4—H4A	120.5	O12—Cl1—O11	110.31 (17)
C14—C15—C16	118.6 (2)	O14—Cl1—O11	108.86 (14)
C14—C15—H15A	120.7	O12—Cl1—O13	108.75 (13)
C16—C15—H15A	120.7	O14—Cl1—O13	109.36 (13)
N4—C13—C14	121.8 (2)	O11—Cl1—O13	109.66 (16)
N4—C13—H13A	119.1	O1W—C1W—H1WA	109.5
C14—C13—H13A	119.1	O1W—C1W—H1WB	109.5
N3—C8—C9	122.6 (2)	H1WA—C1W—H1WB	109.5
N3—C8—C7	115.4 (2)	O1W—C1W—H1WC	109.5
C9—C8—C7	121.9 (2)	H1WA—C1W—H1WC	109.5
C10—C11—C12	119.5 (2)	H1WB—C1W—H1WC	109.5
C10—C11—H11A	120.3	C1W—O1W—H1W	108.2

### Hydrogen-bond geometry (Å, °)

**Table:**

*D*—H···*A*	*D*—H	H···*A*	*D*···*A*	*D*—H···*A*
O1W—H1W···O13^i^	1.03	1.92	2.916 (3)	160

Symmetry codes: (i) *x*−1, *y*, *z*.
